# On the Stability of Reported Pregnancy Intentions from Pregnancy to 1 Year Postnatally: Impact of Choice of Measure, Timing of Assessment, Women’s Characteristics and Outcome of Pregnancy

**DOI:** 10.1007/s10995-019-02748-x

**Published:** 2019-06-19

**Authors:** J. A. Hall, J. Stephenson, G. Barrett

**Affiliations:** Research Department of Reproductive Health, UCL EGA Institute for Women’s Health, London, UK

**Keywords:** Pregnancy intention, Measurement, Stability, Reliability, London Measure of Unplanned Pregnancy, Demographic and Health Survey, Pregnancy outcome

## Abstract

**Objectives:**

Retrospective, cross-sectional estimates of pregnancy intention, as used in the Demographic Health Survey (DHS), are the global norm. The London Measure of Unplanned Pregnancy (LMUP) is a newer, psychometrically validated measure which may be more reliable. This paper assesses the reliability of the LMUP and the DHS question over the first postnatal year and explores the effects of maternal characteristics or pregnancy outcome on reported pregnancy intention.

**Methods:**

We compared the test–retest reliability of the LMUP (using the AC coefficient) and DHS question (using the weighted Kappa) over the first postnatal year using data from Malawian women. We investigated the effect of maternal characteristics and pregnancy outcome using t-tests, Chi squared or Fisher’s exact tests, and calculated odds ratios to estimate effect size.

**Results:**

The DHS question was associated with a statistically significant decrease in the prevalence of unplanned pregnancies from 1-to-12 months postnatally; the LMUP was not. The LMUP had moderate to substantial reliability (0.51–0.66); the DHS had moderate reliability (0.56–0.58). The LMUP’s stability was not related to any of the factors examined; the stability of the DHS varied by marital status (p = 0.033), number of children (p = 0.048) and postnatal depression (p < 0.001). Both underestimated unintended pregnancy postnatally vis-à-vis the LMUP in pregnancy.

**Conclusions for Practice:**

The LMUP is a more reliable measure of pregnancy intention than the DHS in the first postnatal year and does not vary by maternal characteristics or pregnancy outcome. The LMUP should become the gold-standard for measuring pregnancy intention and should be collected in pregnancy or at the first postnatal opportunity.

## Significance Statement

There is growing evidence that current estimates of unintended pregnancy are inaccurate. We show that postnatal assessments of pregnancy intention underestimate unintended pregnancy compared to assessment during pregnancy. The single Demographic and Health Survey (DHS) question is less reliable than the six question, validated London Measure of Unplanned Pregnancy (LMUP). Furthermore, the stability of pregnancy intention estimates using the DHS-question vary by maternal characteristics and some pregnancy outcomes whereas the LMUP estimates do not. For the most accurate measurement of pregnancy intention, we recommend that the LMUP replace the DHS question and that it is used during pregnancy or at the first postnatal opportunity.

## Background

Unplanned pregnancies are of societal interest for a number of reasons, including assessing unmet need for family planning, understanding population level fertility and service requirements, women’s empowerment and agency and the achievement of sexual and reproductive health rights (Joyce et al. 2000; Yeatman and Sennott 2015). Unplanned pregnancies have also been associated with adverse maternal, perinatal and child health outcomes, such as low birth weight, pre-term birth, maternal depression and child development (Gipson et al. 2008; Tsui et al. 2010; Shah et al. 2011; Fisher et al. 2012; Hall et al. 2017). Furthermore, they are of interest because of the significant personal, emotional, financial, physical, psychological and social costs for women and their families (Gipson et al. 2008), and because of the missed opportunities for preconception care and the prevention of unplanned pregnancy (Hall et al. 2016; Stephenson et al. 2018).

Internationally, and in countries like Malawi where this study was conducted, the standard way to assess the prevalence of unplanned pregnancy is in Demographic and Health Surveys (DHS). These are nationally-representative household surveys collecting data on population, health, and nutrition conducted in low- and middle-income countries (The DHS Program). The DHS question on pregnancy intentions asks women to think back up to 5 years to the time they last got pregnant and describe their pregnancy as wanted then (intended), later (mistimed) or not at all (unwanted), with ‘mistimed’ and ‘unwanted’ combined to estimate ‘unintended’. A similar approach is taken by the National Survey of Family Growth (NSFG) and other surveys in the USA.

Using retrospective measures of pregnancy intention gives rise to recall bias given the time that has elapsed between the period of interest (preconception) and the time of the assessment (up to 5 years after birth in the DHS). In addition, there is a significant potential for ex-post rationalization (Rosenzweig and Wolpin 1993; Smith-Greenaway and Sennott 2016) i.e. for the outcome of the pregnancy to affect the reported intention. There is also a risk of social desirability bias as women may be reluctant to describe their child as having been unintended. Several studies have tried to investigate this phenomenon. Two studies (in the USA and India), using the DHS/NSFG style of assessment of pregnancy intention, compared reports of pregnancy intention during pregnancy with reports (on the same pregnancy) after birth (Joyce et al. 2000; Joyce et al. 2002; Koenig et al. 2006). Both studies found that pregnancies classed as unintended during pregnancy tended to shift to be reported as more intended after birth. A small analysis in the UK, comparing women’s reports to health professionals during pregnancy and after birth, produced similar findings (Everett 1991). Three further studies (in the USA and Morocco) assessed women’s post-birth reports of pregnancy intention for the same pregnancy at two postnatal time points, with 3–5 years between comparisons (Westoff 1980; Bankole and Westoff 1998; Guzzo and Hayford 2014). Two studies again found a tendency to report greater intention at the later assessment (Westoff 1980; Bankole and Westoff 1998). The only study to find the opposite was with a slightly different sample; young (18–24) women in the USA who were reporting on their first pregnancy (Guzzo and Hayford 2014).

The limitations of survey questions, such as the DHS, to assess pregnancy intention have been increasingly discussed (Westoff and Ryder 1977; Brown and Eisenberg 1995; Bankole and Westoff 1998; Bachrach and Newcomer 1999; Stanford et al. 2000; Barrett and Wellings 2002; Joyce et al. 2002; Santelli et al. 2003) and a psychometrically validated tool, the London Measure of Unplanned Pregnancy (LMUP) has been developed (Barrett et al. 2004). The LMUP measures the degree of pregnancy intention on an ordinal scale from zero (most unplanned) to 12 (most planned) on the basis of answers to six questions covering contraception, timing of pregnancy, desire, intention, partner discussions and preconception preparations (Barrett et al. 2004; LMUP 2019). The multi-item LMUP allows women to express ambivalence, and for their desires and actions to appear incongruent, does not assume that women’s childbearing intentions are fully formed, and can be used regardless of pregnancy outcome. For prevalence estimates LMUP scores of 0–3 can be considered unplanned pregnancies, 4–9 as ambivalent and 10–12 as planned pregnancies. The LMUP has been validated in 11 languages (Rocca et al. 2010; Morof et al. 2012; Hall et al. 2013; Roshanaei et al. 2015; Borges et al. 2016; Almaghaslah et al. 2017, Habib et al. 2017; Goossens et al. 2018) with further evaluations ongoing. Given its robust development and evaluation, it is expected that such a tool would measure intentions more accurately, and may therefore be more stable, than a dichotomizing question, such as the DHS question.

Evidence on the longer-term reliability of the LMUP is scarce. In the LMUP development study, the weighted kappa for the pregnancy versus postnatal test–retest was 0.86, showing excellent agreement (Barrett et al. 2004). However, the only two further studies to conduct pregnancy versus postnatal test–retests have found considerably lower weighted kappas: 0.43 (moderate stability) among mothers from Bangalore, India, again with a slight shift towards the reporting of more planned pregnancies postnatally (Rocca et al. 2010); and 0.55 (moderate stability) among lower-income mothers from San Francisco, USA, with no significant increase or decrease in LMUP scores between administrations (Morof et al. 2012).

As well as the passage of time, other factors may be associated with the reliability of assessments of pregnancy intention. These include socio-demographic factors, e.g. age, education and marital status; obstetric factors, e.g. the number of children a woman has; and the potential effect of pregnancy outcome and postpartum factors, such as miscarriage, stillbirth, child morbidity and mortality and postnatal depression. There are no published data on how these factors may affect the LMUP but there is evidence that the reliability of DHS-style categorisations of pregnancy intention is associated with factors such as marital status, ethinicity, age, educational attainment and parity as well as child morbidity and mortality (Joyce et al. 2000; Koenig et al. 2006; Smith-Greenaway and Sennott 2016).

Given that retrospective measures of pregnancy intention are used for planning and evaluation purposes at national and international levels, it is vital that we investigate their reliability i.e. to what extent they are affected by the passage of time, women’s characteristics and outcome of pregnancy. This study uses data from a cohort of 4244 pregnant women in Mchinji District, Malawi who were followed up for up to 1 year after the end of their pregnancy (Hall et al. 2016). At the time of the study the median age at first birth in Malawi was 19, the total fertility rate was 5.7 children per woman and 45% of pregnancies were described as unwanted or mistimed (Malawi DHS 2010). This paper aims to: assess the reliability of the LMUP from pregnancy to 1 year postnatally; assess the reliability of the DHS from 1 to 12 months postnatally; and investigate whether maternal characteristics or pregnancy outcome are associated with the stability of pregnancy intention as reported by either LMUP or DHS in the first six postnatal months. This information is used to make recommendations as to which measure should be used and at what time point for the most accurate measure of pregnancy intention.

## Methods

Twenty-five areas were randomly selected from a pre-existing sampling frame of 49 geographical areas of approximately equal population size covering the whole of Mchinji District, Malawi (Lewycka et al. 2013). The 25 areas were grouped into Zones 1, 2 and 3. Pregnant women were identified using a community-based surveillance system and recruited to the cohort between March and December 2013. They were visited, consented and interviewed at their home by a local data collector. Women were eligible for inclusion if they were living in the study area, were at any stage of pregnancy, were aged 15 or over and gave consent. No incentive to participate in the study was provided. The pregnancy interview collected baseline socio-demographic and obstetric history data as well as the validated Chichewa version of the LMUP (Hall et al. 2013).

Women were followed up at home after the end of the neonatal period, with a visit targeted between 6 and 8 weeks’ postnatally, when data were collected on the pregnancy, pregnancy outcome, and the health of the mother and baby for the first 28 postnatal days. Women were also visited at 6 and 12 months’ postnatally, however, due to the rolling nature of recruitment, not all women were eligible for both 6 and 12 months visits before the end of data collection in August 2014.

The Chichewa LMUP was conducted during pregnancy and postnatally; the DHS question on pregnancy intention was only asked postnatally. There was previous evidence of the potential for question order to influence responses on pregnancy intention questions (Kaufmann et al. 1997). In order to explore the possible effect of question order on reported pregnancy intention in our study, we asked the LMUP and DHS questions in a different order postnatally at Zone level. In Zone 1 women were asked the LMUP, then about their prenatal care, then the DHS question. In Zone 2 this was reversed and in Zone 3 women were only asked the DHS question. We found no effect of question order once baseline socio-demographics were controlled for (Hall et al. 2018). Therefore, in this paper our analyses were carried out on all available data (Zones 1–2 for the LMUP analyses; Zones 1–3 for the DHS analyses). For the LMUP, we restricted our analyses to women who were interviewed at 6 months’ gestation (the mean and median gestation of women interviewed), at 1 to 2 months’ postnatally and at 6 and 12 months postnatally (the intended visit times). As we did not ask the DHS question during pregnancy, this analysis was only restricted to women interviewed at the intended postnatal visit times.

We used the non-parametric test for trend (an extension of Kruskall-Wallis) to look at the trend in LMUP score from pregnancy to 12 months postnatally and in DHS categorisation from 1–2 to 12 months postnatally. To assess the test–retest reliability of the LMUP we used the AC coefficient with ordinal weighting (Gwet 2008) to avoid the Kappa paradox of low Kappa despite high agreement which is exacerbated by the 13-point categorisation of the LMUP (Cicchetti and Feinstein 1990; Feinstein and Cicchetti 1990), and for the DHS, which only has three categories, we used Cohen’s kappa (Cohen 1960). We compared the 1–2 months postnatal interview to the pregnancy (for the LMUP) and the 6 and 12 month to the 1–2 months postnatal interview (for the DHS and LMUP). According to Landis and Koch (1977), values of coefficients of agreement can be categorised as follows: 0–0.20 slight, 0.21–0.40 fair, 0.41–0.60 moderate, 0.61–0.80 substantial, and 0.81–1 as excellent agreement. We also report expected and actual agreement.

We assessed the stability of the LMUP score by creating a binary variable for a change in the score, coded zero where the retest LMUP score was within one point of the test LMUP score (no change) and coded one where there was a change of more than one point in either direction. This was used to conduct t-tests for the continuous variables (age, years of education, socio-economic status (SES) score, number of live children, birthweight and postnatal depression score [measured using the validated Chichewa version of the World Health Organization’s Self-Reporting Questionnaire (SRQ) (Harding et al. 1980; Stewart et al. 2009)]). This is a screening tool for anxiety and depression consisting of 20 yes/no questions where each ‘yes’ answer increases the likelihood of depression/anxiety. Where differences were observed, we conducted logistic regression to estimate effect size. Due to the small number of miscarriages, stillbirths and neonatal deaths, and the considerable risk of misclassification between them as these were based on maternal report, we combined these outcomes into one composite adverse outcome variable for all analyses. We conducted Chi squared or Fisher’s exact tests for the binary and categorical variables (adverse pregnancy outcome and marital status). We examined the stability of the LMUP from pregnancy to 1–2 months postnatally and from 1–2 to 6 months postnatally. We conducted the same analysis for DHS stability, using a binary variable coded zero for no change in DHS categorisation and one where the categorisation of the pregnancy had changed. As there was no DHS assessment during pregnancy we only examined the stability of the DHS from 1–2 to 6 months postnatally. We only considered stability at 6 months as the number of women followed up at 12 months for the LMUP was too small for these analyses to be sufficiently powered.

### Ethical Statement

The study from which these data were drawn was approved by the UCL Research Ethics Committee and the College of Medicine Research Ethics Committee at the University of Malawi, reference numbers 3974/001 and P.03/12/1273 respectively. All participants gave written informed consent to take part in this research.

## Results

5887 women were identified as potentially eligible for the main study. 272 had migrated and 1329 were not eligible (usually because they had given birth) leaving 4286 women. Of these, 42 did not consent; a refusal rate of < 1%, leaving 4244 pregnant women. The cohort has been described elsewhere (Hall et al. 2016) but, in brief, women were aged 15–49, and tended to be married (> 90%) with low levels of education (86% had no/primary education only). The predominant religion was Christian and most women were from the Chewa tribe. Women reported up to 15 pregnancies (median three) and 12 previous births (median two).

**Fig. 1:**
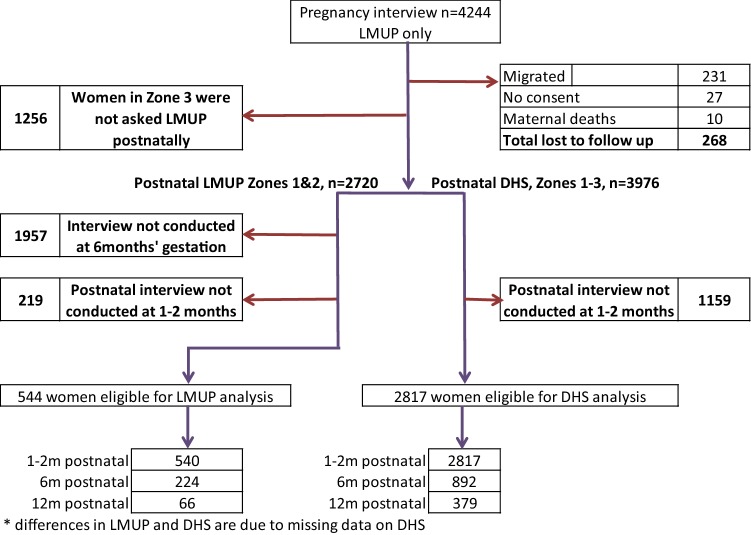
Flow chart of recruitment and follow-up of women in the cohort

The number completing follow up at each time point is shown in Fig. 1. Due to the rolling nature of recruitment, not all women reached 6 or 12-month follow-up before the end of the project. As the original study was focused on neonatal outcomes, the cohort closed when the last women recruited completed this milestone, in July 2014 so only those women whose babies had been born early in the study, which started data collection in March 2013, had the potential to reach 12 months. Also we have restricted our analyses to data collected in the intended postnatal months. There were no significant differences in the age, education, SES, marital status or number of live children of women who were interviewed at 6 months’ gestation compared to those interviewed at other gestations. Nor were there any differences in the sample of women interviewed at 1–2 or 6 months postnatally compared to those who were not. For the 12 month LMUP sample, the 66 women included were slightly younger (22.8 vs. 25.0 years, p = 0.01) and consequently had fewer children (1.23 vs. 1.79, p = 0.02). There were no significant differences in the 12 month DHS sample.

### Stability of LMUP from Pregnancy to 12 Months Postnatally

Table 1 shows the descriptive statistics of the LMUP score in pregnancy and at each postnatal follow up, as well as the mean and median differences, expected and actual agreement and AC coefficient. There was an increase in LMUP scores over this time, from 7.16 in pregnancy to 8.07 at 1–2 months postnatally to 8.42 at 12 months. The test for trend was significant from pregnancy to 12 months postnatal (p = 0.003), and non-significant between 1–2 and 12 months postnatally (p = 0.239). The LMUP had moderate to substantial agreement from pregnancy to 12 month’s postnatally, substantial agreement between the 1–2 and 6 months postnatal measures, and moderate agreement between the 1–2 and 12 months postnatal measures. The changes in LMUP score corresponded to an increase in the prevalence of intended pregnancies from 45.2% during pregnancy to 59.6% at 1–2 months and 65.3% at 12 months postnatally.

**Table 1:** Antenatal LMUP score and change from pregnancy to 12 months postnatally and from first postnatal to 12 months postnatally in Zones 1 and 2

LMUP Score		Postnatal		
	Pregnancy	1–2 months	6 months	12 months
Number	544	540	224	66
Mean	7.16	8.07	7.76	8.42
Standard deviation	3.89	3.72	3.99	4.27
Median	9	10	10	10
Inter-quartile range	3, 11	4, 11	3, 11	5, 12
Mean difference to pregnancy score		0.93	0.82	0.94
Median difference to pregnancy score		0	0	1
Expected agreement with pregnancy score		79%	78%	77%
Actual agreement with pregnancy score		92%	89%	90%
AC to pregnancy score		0.64	0.54	0.60
Mean difference to first postnatal score		–	− 0.03	− 0.12
Median difference to first postnatal score		–	0	0
Expected agreement with first postnatal score		–	78%	79%
Actual agreement with first postnatal score		–	92%	87%
AC to first postnatal score		–	0.66	0.51

While most women (61%) did not change their LMUP score from pregnancy to 1–2 months postnatally, 11% (n = 61) decreased and 28% (n = 150) increased their score by more than one point. Women with a lower LMUP score were more likely to increase their score at 1–2 months postnally; for every one point increase in LMUP score during pregnancy women had 0.86 (95% CI 0.80, 0.93) the odds of increasing their LMUP score by more than one point compared to women who did not change or who decreased their score.

The only factor affecting the stability of the LMUP between the pregnancy and 1–2 month postnatal measurement was number of live children (p = 0.001) (Table 2); for every additional child, women had 1.18 (95% CI 1.06, 1.30) the odds of increasing their LMUP score. There was no difference by postnatal depression or adverse pregnancy outcome.

**Table 2:** Differences in mother’s characteristics and pregnancy outcome by whether or not their LMUP score changed by more than one point from pregnancy to 1–2 months postnatal visit and between 1–2 and 6 months postnatal visits

	Pregnancy to 1–2 months postnatal measurement			
	LMUP score did not change (n = 329) mean	LMUP score did change (n = 211) mean	Total mean	*p* value
Mother’s age (years)	24.5	25.3	24.8	0.187
Mother’s education (years)	5.48	5.36	5.43	0.678
Socio-economic status score	0.006	0.133	0.056	0.406
Number of living children	1.53	2.03	1.73	**0.001**
Birthweight	3.16	3.19	3.17	0.591
SRQ score	2.76	2.32	2.59	0.12

	%	%	Total %	
Married	92.1	92.9	92.4	0.868
Adverse pregnancy outcome	4.26	6.64	5.19	0.237

	1–2 to 6 months postnatal measurement			
	LMUP score did not change (n = 151) mean	LMUP score did change (n = 72)	Total mean	*p* value
Mother’s age (years)	24.5	25.5	24.8	0.246
Mother’s education (years)	5.81	5.18	5.61	0.162
Socio-economic status score	0.03	0.44	0.16	0.09
Number of living children	1.48	1.82	1.59	0.159
LMUP score in pregnancy	7.27	6.25	6.94	0.072
Birthweight	3.16	3.09	3.14	0.419
SRQ score	2.54	3.02	2.70	0.277

	%	%	Total %	
Married	90.1	90.3	90.1	0.584
Adverse pregnancy outcome	4.64	1.39	3.59^a^	0.442

^a^Note only eight adverse outcomes

Bold value is statistically significant (*p* < 0.05)

There were no differences in the stability of the LMUP between the 1–2 and 6 months postnatal measurements on any maternal characteristic or outcome.

### Stability of the DHS from 1 to 12 Months Postnatally

There was an increase in the proportion of pregnancies reported as intended on the DHS question from the first to twelfth postnatal month, from 61.9 to 67.6% (Table 3). This trend was statistically significant (p = 0.031). 27.4% of women changed category between 1–2 and 6 months postnatally, with 16.8% increasing their reported intention and 10.4% decreasing it. The DHS has moderate agreement across the first postnatal year.

**Table 3:** Change in the DHS categorisations of pregnancy intention across the first postnatal year

	Postnatal DHS		
	1–2 months [n (%)]	6 months [n (%)]	12 months [n (%)]
Intended	1736 (61.9)	579 (64.8)	256 (67.6)
Mistimed	589 (21.0)	156 (17.5)	66 (17.4)
Unwanted	480 (17.1)	159 (17.8)	57 (15.0)
Total	2805 (100)	894 (100)	379 (100)
Expected agreement		69.7%	69.1%
Actual agreement		87.2%	83.2%
Kappa		0.58	0.46

There were significant differences in the stability of the DHS from the 1–2 to 6 months postnatal assessment by several variables (Table 4). These were number of live children (odds of changing DHS category increase by 1.08 (95% CI 1.00, 1.17) for each additional child), risk of postnatal depression (odds of changing DHS category increase by 1.10 (95% CI 1.05, 1.16) for each additional point on the SRQ, indicating a higher risk of depression), and marital status (unmarried women had 1.73 times the odd of changing (95% CI 1.04, 2.89)).

**Table 4:** Differences in mother’s characteristics and pregnancy outcome by whether or not their DHS categorization changed between 1–2 and 6 months postnatal visits

	1–2 to 6 months postnatal visit			
	DHS did not change (n = 657) mean	DHS did change (n = 235) mean	Total mean	*p* value
Mother’s age (years)	25.0	25.8	25.2	0.110
Mother’s education (years)	5.42	5.40	5.40	0.908
Socio-economic status score	0.10	0.89	0.10	0.932
Number of living children	1.79	2.07	1.87	**0.048**
Birthweight	3.13	3.16	3.14	0.520
SRQ score	2.25	3.09	2.47	**<0.001**

	%	%	Total %	
Married	93.3	88.9	92.2	**0.033**
Adverse pregnancy outcome^a^	3.35	2.98	3.25	0.784

^a^Note only 29 adverse outcomes

Bold values are statistically significant (*p* < 0.05)

### Comparison of the LMUP and DHS Estimates of the Prevalence of Intended Pregnancy

The DHS finds a statistically significantly higher prevalence of intended pregnancy than the LMUP at every postnatal time point within the same women (Table 5). When compared with the LMUP estimate during pregnancy of 45.2% intended pregnancies (LMUP score 10–12), the DHS at 12 months estimates 84.2% pregnancies as intended (p < 0.001), significantly underestimating the prevalence of unplanned pregnancy when compared with the LMUP assessment during pregnancy.

**Table 5:** Comparison of the percentage of pregnancies classified as intended on the LMUP and DHS at each time point

	Percentage (n) of intended pregnancies		
	LMUP	DHS	*p* value
During pregnancy	45.2 (544)	–	
1–2 months postnatal	59.6 (540)	65.9 (539)	**0.034**
6 months postnatal	58.4 (452)	69.5 (429)	**0.001**
12 months postnatal	65.3 (121)	84.2 (101)	**0.001**

Bold values are statistically significant (*p* < 0.05)

## Discussion

Our research is the first to investigate the long-term reliability of the LMUP in a pre/post birth situation at multiple time points up to the end of the first postnatal year. It is also the first to compare the LMUP and DHS and to investigate the effect of miscarriage, stillbirth, and neonatal mortality, low birth weight, or postpartum depression on the stability of reported pregnancy intention using any measure.

The fact that the reliability of the LMUP was not significantly affected by any of the pregnancy outcomes we considered, when the DHS was affected by the SRQ score, a marker for anxiety and depression, and has previously been shown to be affected by child morbidity and mortality (Smith-Greenaway and Sennott 2016) suggests that the issue of post-hoc rationalisation may be less important when pregnancy intention is assessed with a psychometric measure.

Our analyses demonstrated that both the LMUP and the DHS show a general increase in the reported degree of intention over the first postnatal year. This was not statistically significant for the LMUP but was for the DHS. This is consistent with previous research that has shown an increase in reported intention over time (Westoff 1980; Bankole and Westoff 1998; Joyce et al. 2000; Joyce et al. 2002; Koenig et al. 2006). Both the DHS and the LMUP used at 12 months postnatally led to an underestimate of the proportion of unplanned pregnancies by between 20 and 39%, when compared to the LMUP assessment during pregnancy. What happens beyond 1 year is not known for the LMUP; previous studies using repeat measures of the DHS and similar questions have shown that estimates at 3 to 5 years are subject to the same issue (Westoff 1980; Bankole and Westoff 1998; Joyce et al. 2002; Koenig et al. 2006).

Furthermore, the stability of the LMUP between pregnancy and the 1–2 months postnatal assessment was only affected by the number of live children and was not affected by any factors between the 1–2 and 6 months postnatal assessments. Conversely, women with more children, married women and women who scored more highly on the SRQ measure of anxiety and depression were all more likely to change categories on the DHS. These findings are in keeping with previous literature. In the USA, Joyce et al. (2000) used data from the National Longitudinal Survey of Labor Market (which asked a question very similar to the DHS) to investigate determinants of a change in reported intention. They found that women who changed from intended in pregnancy to unintended after birth were more likely to be black, never married, with a lower family income and a lower score on the Armed Forces Qualification test (all p < 0.05). Another study in India, using the DHS question, found that the stability of intentions varied with maternal age and number of living children (Koenig et al. 2006).

Despite its limitations, the DHS question on pregnancy intention has been used for decades and has provided useful information. Unlike the DHS question, the LMUP is a psychometrically validated measure of pregnancy intention. It was developed following extensive qualitative work to map the construct of pregnancy intention, with questions developed, field tested and refined to ensure that the full construct is represented in the measure (Barrett et al. 2004). The LMUP’s six questions capture a multidimensional assessment of pregnancy intention, covering women’s behaviours, context and desires, whereas the DHS question only asks about the timing of the pregnancy in relation to childbearing plans. While the LMUP is longer than the DHS, self-completion takes < 2 min.

Due to the methodology of its development, the LMUP potentially overcomes some of the limitations of the DHS question. Our data would seem to support this conclusion, hence we recommend replacing the DHS question with the LMUP. There may be resistance to this due to the challenges involved in making the change and concerns about measuring trends over time. However, the recent change from a one- to two-question sequence in the DHS shows that change is possible. Even this change could cause disruption to measuring trends over time by potentially introducing confusion between mistimed and unwanted births (Bearak et al. 2018; Maddow-Zimet and Kost 2019). In 2015 an expert meeting convened by the ‘Strengthening Evidence for Programming on Unintended Pregnancy Research Programme Consortium' recognised that the LMUP had many strengths over other current measures but concluded that there was insufficient evidence to recommend the LMUP at that time as there were only three published validation studies (Population Council 2015). The LMUP is now being used in research globally; with 11 published validations and 13 as yet unpublished evaluations in diverse settings there is now sufficient evidence to support the introduction of the LMUP as the global standard measure of unintended pregnancy.

### Limitations

This study uses data from pregnant women in rural Malawi. While the study sample was considered representative of Malawi in general (Hall et al. 2016) our findings may not be generalizable to settings with, for example, lower fertility rates. However, as we have shown, our findings are in keeping with the few studies from both high and middle-income countries on this topic (Joyce et al. 2000; Koenig et al. 2006; Smith-Greenaway and Sennott 2016). We were unable to assess the effect of infant mortality on the stability of intentions due to the small number of deaths.

Although we assessed pregnancy intention during pregnancy with the LMUP, this is still a retrospective assessment as we are primarily interested in intentions prior to pregnancy. Few studies have compared pre- and postconception estimates of pregnancy intentions given the difficulties of doing so. However, Yeatman and Sennott (2015) compared different ways of assessing unintended pregnancy, including prospective, retrospective and time-varying measures, in over 1000 young Malawian women who were interviewed every 4 months over 2½ years. They also found that retrospective assessment tended to underestimate unintended pregnancies and they showed that the most consistent estimate of unintended pregnancy was between the most recent prospective (i.e. preconception) estimate and the estimate during pregnancy.

## Conclusion

Our findings suggest that the LMUP is a more reliable measure of pregnancy intention than the DHS. We have shown that the use of postnatal assessments, either with the LMUP or the DHS, underestimate the prevalence of unplanned pregnancy when compared with assessment in pregnancy. The LMUP has been validated in heterogeneous settings around the world demonstrating its relevance and utility. There is now considerable evidence that the DHS-style questions underestimate unintended pregnancy and are affected by maternal characteristics and pregnancy outcome in a way that the LMUP does not seem to be. This leads us to recommend the LMUP to replace DHS-style questions as the gold standard for the measurement of pregnancy intentions worldwide. To increase accuracy further, we recommend that the LMUP should be collected during pregnancy, for example at prenatal and abortion services, or at the earliest postnatal opportunity, e.g. the 1-week check.

As long as the prevalence of pregnancy intention is assessed postnatally and using DHS-style questions, we will continue to underestimate the scale of the problem of unplanned pregnancy and consequently the issue will not receive the attention, and funding, it requires. Preventing unplanned pregnancies is fundamental to improving women’s sexual and reproductive health and rights and requires investment in women’s education and agency, as well as sexual and reproductive health services, so that they have the ability to decide if and when to have children and to act on these intentions.
